# Developing a Case-Based Blended Learning Ecosystem to Optimize Precision Medicine: Reducing Overdiagnosis and Overtreatment

**DOI:** 10.3390/healthcare6030078

**Published:** 2018-07-10

**Authors:** Vivek Podder, Binod Dhakal, Gousia Ummae Salma Shaik, Kaushik Sundar, Madhava Sai Sivapuram, Vijay Kumar Chattu, Rakesh Biswas

**Affiliations:** 1 Department of Internal Medicine, Tairunnessa Memorial Medical College, Gazipur 1704, Bangladesh; drvivekpodder@gmail.com; 2 Division of Hematology/Oncology, Medical College of Wisconsin, Milwaukee, WI 53226, USA; bdhakal@mcw.edu; 3 Department of Internal Medicine, Kamineni Institute of Medical Sciences, Narketpally 508254, India; drshaiksalma@gmail.com; 4 Department of Neurology, Rajagiri Hospital, Chunanangamvely, Aluva 683112, India; skaushik85@gmail.com; 5 Department of Internal Medicine, Dr. Pinnamaneni Siddhartha Institute of Medical Sciences and Research Foundation, Chinaoutapalli 521101, India; madhavasai2011@gmail.com; 6 Department of Paraclinical Sciences, Faculty of Medical Sciences, The University of the West Indies, St. Augustine 0000, Trinidad and Tobago; vijay.chattu@sta.uwi.edu

**Keywords:** overdiagnosis, overtreatment, CBBLE (case-based blended learning ecosystem), case studies, precision medicine, omics driven, low resource setting, high resource setting

## Abstract

Introduction: Precision medicine aims to focus on meeting patient requirements accurately, optimizing patient outcomes, and reducing under-/overdiagnosis and therapy. We aim to offer a fresh perspective on accuracy driven “age-old precision medicine” and illustrate how newer case-based blended learning ecosystems (CBBLE) can strengthen the bridge between age-old precision approaches with modern technology and omics-driven approaches. Methodology: We present a series of cases and examine the role of precision medicine within a “case-based blended learning ecosystem” (CBBLE) as a practicable tool to reduce overdiagnosis and overtreatment. We illustrated the workflow of our CBBLE through case-based narratives from global students of CBBLE in high and low resource settings as is reflected in global health. Results: Four micro-narratives based on collective past experiences were generated to explain concepts of age-old patient-centered scientific accuracy and precision and four macro-narratives were collected from individual learners in our CBBLE. Insights gathered from a critical appraisal and thematic analysis of the narratives were discussed. Discussion and conclusion: Case-based narratives from the individual learners in our CBBLE amply illustrate their journeys beginning with “age-old precision thinking” in low-resource settings and progressing to “omics-driven” high-resource precision medicine setups to demonstrate how the approaches, used judiciously, might reduce the current pandemic of over-/underdiagnosis and over-/undertreatment.

## 1. Introduction

The term “Precision Medicine” was first coined by Clayton Christensen in his book the “Innovator’s Prescription”, published in 2009 [1]. According to the early definition given by the Institute of Precision Medicine, “Precision medicine is targeted, individualized care that is tailored to each patient based on his or her specific genetic profile and medical history” [2].

While the above definitions allow us to assume that precision medicine is focused on meeting patients’ requirements accurately, we need to review the scientific nature of accuracy, precision and their relationship with each other to put things in perspective. This is essential toward optimizing patient requirements and outcomes, minimizing damage to the healthcare ecosystem by reducing under-overdiagnosis and therapy. To quote from Thomas (2014), “The healthcare ‘system’ is now better understood as an ‘ecosystem’ of interconnected stakeholders, each one charged with a mission to improve the quality of care while lowering its cost. To ensure patient safety and quality care while realizing savings, these stakeholders are building new relationships—often outside the four walls of the hospital” [3].

**Figure 1:**
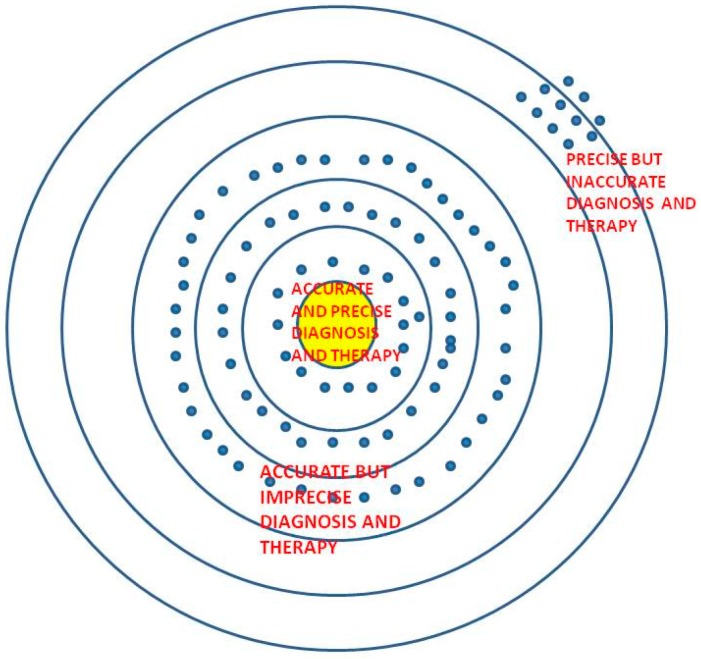
Mapping ‘Precision’ and ‘Accuracy’ in Medicine (Accuracy—achieving patients’ requirement of best outcome, and Precision—the narrowest path to achieve it).

**Figure 2:**
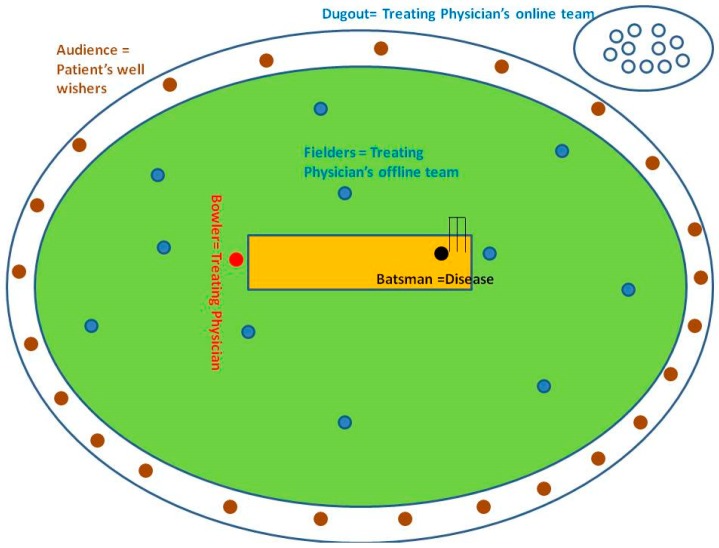
Illustrates the analogy of healthcare ecosystem functioning in the form of a cricket match.

We illustrate current concepts borrowed from existing scientific literature around accuracy and precision with Figure 1 and Figure 2. We have modified the figure in reference to paper [4] to offer a fresh perspective on accuracy-driven “precision medicine”, which is an age-old tool for physicians, currently augmented by technology.

### 1.1. Precision versus Accuracy

We illustrate the above concepts with micro case studies below:

“An elderly patient from a country endemic with tuberculosis presented with a chronic cough and weight loss. A lung pathology was detected on imaging that was not amenable to further biopsy efforts as a result of unavailable resources. He was started on empirical treatment for tuberculosis after sending a sputum for acid-fast bacillus (AFB) and culture.”

In tuberculosis endemic countries, physicians often treat empirically for tuberculosis in suspicious lung pathologies, although lung malignancy is a close differential in such situations. In the above patient’s context, physicians were being obviously imprecise in starting treatment for tuberculosis empirically even when the tuberculosis bacilli was undetectable. This is an acceptable standard practice with established protocols for treating sputum-negative tuberculosis utilized globally by many countries that are endemic for tuberculosis.

Now to illustrate the concepts further, the above case may have the following mentioned outcomes:

(a) The elderly patient’s sputum comes out to be positive and once he is begun on antitubercular therapy, he recovers. His cough subsides and weight improves and his sputum culture report that comes after 6 weeks also turns out to be positive for tuberculosis. This is an example of precise and accurate diagnosis and treatment.
(b) The elderly patient’s sputum turns out to be negative and yet once he is begun on antitubercular therapy, he recovers. His cough subsides and weight improves and his sputum culture report that comes after 6 weeks turns out to be positive for tuberculosis although his initial sputum smear was negative. This would be an example of initially imprecise, but finally accurate outcomes.
(c) The elderly patient’s sputum turns out to be negative and a cartridge-based nucleic acid amplification assay test (CBNAAT) sent at the same time also turns out to be negative for tuberculosis and once he is begun on antitubercular therapy, he does not appear to recover at all. His cough worsens along with his appetite and his weight loss increases. A bronchoscopy with bronchoalveolar lavage is performed and sent again for malignant cytology, AFB, CBNAAT, and culture. His sputum culture report that comes after 6 weeks turns out to be positive for drug-resistant tuberculosis. Although, he receives second-line therapy for his drug-resistant tuberculosis, his condition worsens, and he dies.

The above is an example of a precise approach that still leads to inaccurate patient outcomes. We can be inaccurate in spite of being precise because of the current limitation of information and knowledge that does not always allow us to be accurate. The role of research and learning is to address this limitation and push the boundaries of current knowledge. Precision medicine develops and positively evolves with better research and learning.

(d) The elderly patient’s sputum turns out to be negative for tuberculosis and no further tests are done due to lack of resources. He is put on empirical antitubercular therapy, but he does not appear to recover at all. His cough worsens along with his appetite and his weight loss increases. One day he has a sudden shortness of breath and dies. An autopsy reveals bronchogenic carcinoma and pulmonary embolism.

The above is an example of imprecise and inaccurate outcomes. Here again, the obvious precipitants are low resources that prevent further probing toward a precise diagnosis, leading one to resort to imprecise treatment.

Out of the descriptions of a–d above, let’s focus on (b), which illustrates imprecise approaches to arrive at accuracy. This is also known as the trial and error method in common parlance. Over the past centuries, medicine has often relied on this imprecise trial and error method, particularly on individual patients before it was replaced by the population-based randomized controlled trial approach to collect generalizable average evidence that is often applied on individual patients. The problem with this current approach is that the requirements of the individual at hand may not always match the average requirements.

Nevertheless, modern-day, evidence-based precision demands that the individual is first viewed through the ‘average evidence’ lens. Once the individual’s response to the ‘average’ evidence-based available therapy is suboptimal or the therapy itself is unavailable, a therapeutic trial on the individual can be undertaken as a single subject ‘n of 1′ study design.

Coming back to (b), which is about a situation where empirical therapy is provided to an individual who may have a substantial chance of having an alternative diagnosis, and in (b) that individual turns out to be lucky to have that very diagnosis that was targeted. This imprecise approach may often be overdone in low-resource settings, where one often comes across prescriptions listing out 9–10 medications where the strategy is to target all possible differentials with whatever medicine appears to have the slightest evidence of success. While this form of irrational overuse of medication is a global problem, it appears to be more pronounced in low-resource settings [5]. This problem, a lack of system to make practitioners aware of their follies, is more common in low-resource settings also because of arcane medical education strategies that do not train students in critical appraisal, neither in terms of recognizing nor applying current best evidence. This deficit often stems from the education system’s overreliance on rote memorization based on a curriculum that discourages students to ask questions [6]. One solution to the current curricular conundrum is to create more CBBLEs in every medical college, where patient-centered, evidence-based, self-directed learning is the prime emphasis [7].

Behnke LM et al. has defined overutilization as “*use of unnecessary care when alternatives may produce similar outcomes, results in a higher cost without increased value*” [8]. Overuse has a huge burden on low- and middle-income countries, where much healthcare is provided by ill-regulated, private providers and with fee-for-service. As a result, healthcare costs significantly increase and potentially harm patients through inappropriate interventions.

While our above description of overuse is true for multiple medication overuse in single patients, some single interventions can be scaled rapidly in larger populations. Current global concerns are mounting over inappropriate interventions such as percutaneous coronary intervention (PCI) for patients who are unlikely to benefit.

Brownlee S et al. reported that in an Indian second opinion setting, 55% of recommended PCIs were ill-advised [9]. A study in a tertiary care center in India showed that ST-elevation myocardial infarction (STEMI) patients constituted 55% of all inappropriate elective PCIs. These PCI procedures were performed on totally occluded infarct-related vessels after 12 h of symptom onset. The same study also showed that patients with stable angina with single or double vessel disease, low-risk group and sub-optimal medical therapy constituted 45% of all inappropriate elective PCIs [10]. It has been reported that the prevalence of inappropriate PCI procedures is 3.7% in Korea; 12% in the USA; 14% in Germany; 16% in Italy; 22% in Israel and 20% in Spain [9].

Over the last decade, we have adopted an evidence-based precision medicine approach that enables utilizing the best available evidence toward optimizing care for individual patients. Our individual patient requirements have led us to adopt a blended learning platform to enable an informational support for our patients. It also helps medical students to have a platform to help patients locally while learning from global experts in an online ecosystem [11]. Our online learning ecosystem is a community of computer and mobile users comprised of medical students, their physician teachers, other health professionals and patients and their relatives, each one of whom provide inputs (in terms of case-based information) to this ecosystem through devices. They receive learning feedback on the same cases such that their learning outcomes can be potentially translated into patient outcomes. This system, labeled “user-driven healthcare” (UDHC), is not restricted to our CBBLE, but represents an evolving global “phenomenon”. Here “improved healthcare is achieved through concerted collaborative learning between multiple users and stakeholders, primarily patients, health professionals and other actors in the caregiving collaborative network across a web interface”. This has been described in detail elsewhere [12,13,14,15,16,17,18].

### 1.2. Case-based Blended Learning Ecosystem (CBBLE): Current Case Studies and Implications for Precision Medicine

“The idea of sharing and learning around patients has been alive since the beginning of medicine, when physicians would present their cases to a large audience to primarily learn from the inputs of other physicians.” With the invention of the printing press, instead of restricting themselves to verbal face-to-face case presentations, many physicians published their cases in journals and slowly the medical fraternity started naming those published diseases after their first authors. “In this way, case reporting became a gainful activity not only in terms of scientific advancement towards patient benefits, but also as an important instrument of physician fame.” We have utilized this case reporting model to help our patients and to train our medical students about disease and patient experience [18,19].

By reporting cases, this model allows more engagement both from patients and medical students to reach a precise and accurate diagnosis, and also helps as an educational tool.

Our healthcare learning ecosystem is currently based offline in the Kamineni Institute of Medical Sciences, and this offline base keeps shifting with the various university locations in India where our corresponding authors are based for varying numbers of years. The online component of this blended learning ecosystem began on email groups, and then shifted to social media groups such as “Tabula Rasa” [14]. It currently exists in WhatsApp groups with a global audience of medical students and physicians. Gauging by the mentions in our past publications that have flowered through offline inputs processed further online, the students and faculty driving our online discussions range from universities in the US; India; Oxford, UK; Ontario, Canada; Montpellier, France; and Monash, Australia; as well as in the Maldives, Nepal, and Bangladesh.

What follows is a series of case narratives by the physicians in our case-based learning ecosystem and our critical appraisal of them through the “precision lens”. We have previously published detailed patient narratives of online patient users of our ecosystem [18], and this article focuses on the physician narratives to provide a qualitative perspective of our workflow.

## 2. Materials and Methods

These case studies were taken from our “case-based blended learning ecosystem” (CBBLE) and were narrated by the physicians, students, and their senior teachers. We illustrated current concepts of scientific accuracy and precision using two analogies. The first was an age-old analogy of “scientific precision” and the second was a creative analogy comparing the medical endeavor of our CBBLE to a game of cricket where health professional bowlers and fielders try to get the batting disease team out in time to win the match. While this is not far from established analogies of “battling” disease, the cricket stadium offers a detailed perspective of our CBBLE framework. A group of our CBBLE online students visited Kamineni Institute of Medical Sciences (KIMS), Narketpally, where its offline component is currently ongoing. They worked with the team of students, physically managing the patients and developing insights into the nature of precision that enabled further analysis of the narratives obtained from our CBBLE students past and present.

This was done to attain our objective of illustrating the role of accuracy-driven “age-old precision medicine” in the framework of our current CBBLE, parallel to current “OMICS-driven” precision medicine narratives, and how we may create a bridge between the two systems.

## 3. Results

### 3.1. Precision Medicine through a Physician Resident’s Narrative Lens (in Relatively Low-Resource Settings)

SS is a postgraduate resident physician managing critically ill patients, as well as chronic patients in the outpatient department (OPD) as part of her formal residency program in medicine. She also works part of the time on a thesis that involves clinical decision making around management of thyroid disorders. 

What follows is her own narrative of experiences with one recent critically ill patient that she managed onsite, offline along with her other resident colleague as well as other first-year postgraduate colleagues, also known as interns. This was observed in real time by the online members of our CBBLE, who supported with their input once SS shared the patient’s deidentified online record on a blog. This was preceded by a snippet of the patient’s computed tomography (CT) images, as well as a very brief history of her problem. The online record blog link is accessible in [20].

After dinner on my night duty, I rushed to casualty as a patient was brought who needed immediate attention. On going to casualty, I saw that a 60-year-old lady was struggling to breathe and appeared tachypneic. Her % oxygen saturation was only 86% at room air. We had to provide her immediate oxygen support which improved her saturation but she was still tachypneic, and opening her mouth to take in the air, basically struggling to take the air in.

After making sure that her saturation was well maintaining I had called the attenders to take a detailed history. Her complaints were not of recent onset. She had complaints since the past 4 months. The lady had been having complaints of burning micturition since 4 months. She had been visiting the hospital often for the above complaints. This time, she had the same complaints of burning micturition, fever, abdominal pain and decreased urine output.

On asking further the attenders related that she hadn’t passed urine since 3–4 days. So I had immediately asked for a Foley’s catheterization. The moment we had inserted the Foley’s the urine was milky white for the initial few minutes. By this time we had got a few of their old prescriptions which showed a urology op card and the urologist made a diagnosis based upon the history of “thin stream of urine” as stricture urethra. Now everything was falling into place. Her persistent complaints from the past few months, her burning micturition, fever.

We got the necessary investigations done, firstly sent a blood and urine culture as I could sense that she may deteriorate. Sent immediately blood for an arterial blood gas (ABG) analysis which showed a metabolic acidosis thus ruling out any lung pathology leading to shortness of breath. She needed dialysis. Blood counts showed elevated leukocytes. Her urine routine microscopy showed plenty of pus cells. Her ultrasonogram (USG)of the abdomen revealed pyonephrosis with dilated ureters.

Had got her dialysis done the next day. In her Foley’s interestingly we noted some debris. We had a discussion around it as what it could be. But post dialysis though her acidosis resolved she went into hypotension. We had to start her on inotropic support. Had to monitor her blood pressure (BP) closely to tailor the dose accordingly. We were eagerly waiting for her culture reports as the leucocyte count showed an increasing trend. By this time we had got all her previous outpatient cards which not only revealed her past history and procedures done but were also a representation of the case-based workflow of multiple departments in our rural tertiary medical college hospital.

Her previous outpatient cards revealed that her first visit was in December last year. She had first gone to a gynecologist with complaints of burning micturition, whitish discharge per vaginum and fever. Probably the gynecologist had asked her to void and come as they wanted to examine her per vaginally. But this is where we lost track of the information in her outpatient card progress notes. She then landed up in urology OPD where her op card revealed that she had a thin stream of urine. The diagnosis of stricture urethra was made solely based on this and a dilatation procedure was done, she was catheterized with Foley’s and urine culture sensitivity was sent. She was started on antibiotic and asked to turn up after 3 days which she did. She got her Foley’s removed and was prescribed the 1st line antibiotic for urinary tract infection (UTI). The urine culture report was, unseen by the care provider, and unasked by the patients. She was prescribed the same 1st line antibiotic as in the first visit. She was then asymptomatic for the next 2 months. When she developed her current symptoms she turned up in the casualty this time.

The organism was the same since the first report, but sadly the culture reports being unseen she was getting the antibiotic for which the organism was never sensitive. The organism had been evolving since then into a more virulent one.

#### 3.1.1. Analogy-Driven Analysis: Retrospective Notes from the Dugout

We draw below an analogy of our blended ecosystem with a cricket match (Figure 2), where the bowler is the treating physician, along with the rest of the fielders as a part of the treating team. The dugout is the online team sending inputs to the bowler, also known as, the offline treating physician team for taking the wicket of that batsman, also known as, the disease. 

The main aim of the entire offline and the online team is to take an accurate wicket using a precise strategy that minimizes overuse and overtreatment and successfully banishes the disease to the pavilion and accurately meets patient requirements. Precision medicine ensures the narrowest path toward accuracy.

The healthcare professional team, as bowlers and fielders, rushed to the casualty (field) to battle the disease of this patient and win the match to meet the patient requirements within a narrowest path possible to attain precision and accuracy without overdiagnosis and overtreatment (Figure 2).

The postmenopausal woman was prescribed first-line antibiotics for UTI without following the urine c/s reports that precisely pointed toward the sensitivity. This permitted the E. coli to strengthen, as the antibiotic in use was a poor match. Moreover, an estrogen ointment was prescribed for local use, although evidence of efficacy for this intervention for UTI prevention or treatment is inconclusive [21]. Lacking adequate diagnosis and follow-up, she returned to casualty with life-threatening symptoms.

The above is an example of an initial accurate diagnosis managed with an imprecise approach leading to inaccuracy.

Physician resident’s narrative, continued

We got a CT abdomen done for her as the USG abdomen revealed pyonephrosis. We had something very unusual and unexpected in store for us in the CT. She had air pockets in the kidney, in the erector spinae muscle and spinal canal. We decided to change the antibiotic. We were still waiting for the culture report and had started her on a higher antibiotic. But to our dismay, our microbiology department didn’t have the sensitivity checking disc for the antibiotic which we had started her on.

#### 3.1.2. Online Work flow in Parallel with the Offline Component of Our CBBLE

**Figure 3:**
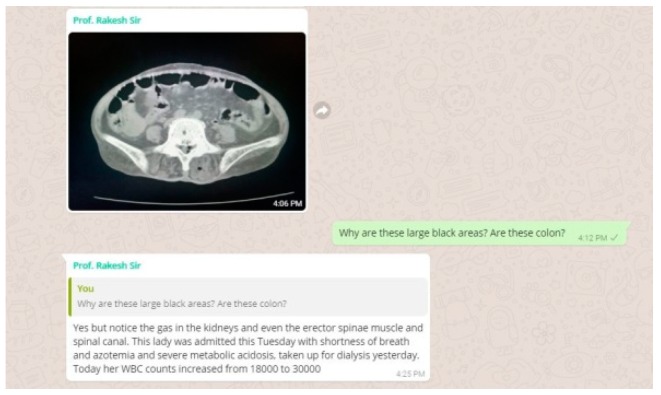
Illustrating the patient data shared online into our CBBLE online network.

**Figure 4:**
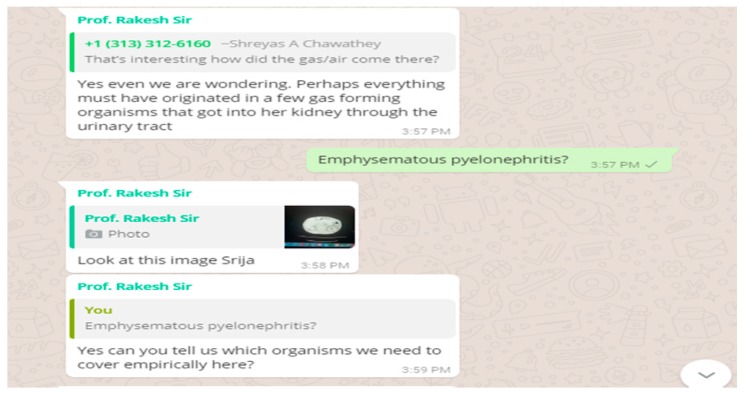
Illustrating the patient data shared online into our CBBLE online network.

**Figure 5:**
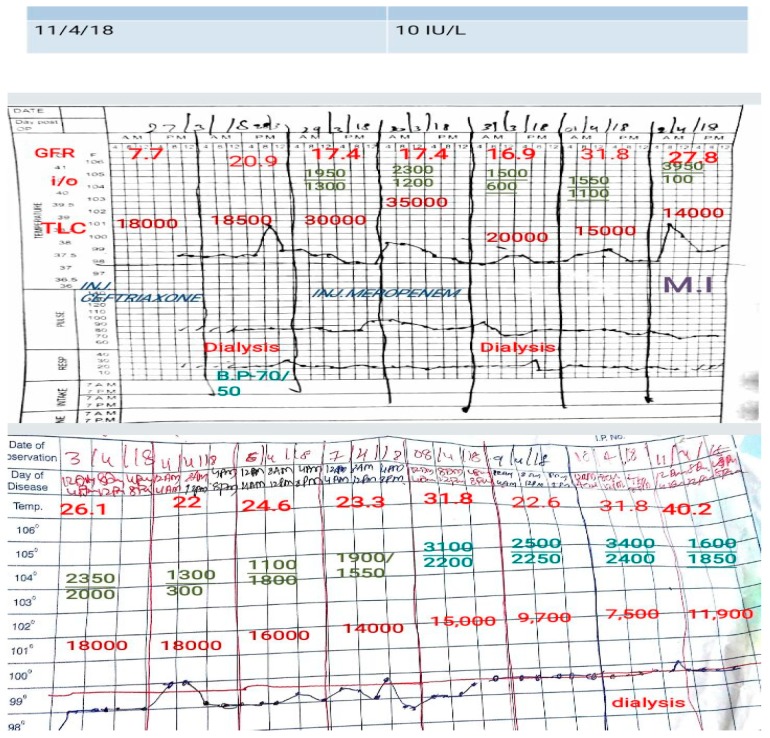
Illustrates the graphical representation of her illness during her stay in the hospital.

At this point, the patient data was shared online in our CBBLE and the online team (analogous to the stadium dugout) swung into action (Figure 3 and Figure 4). This enriched the decision-making capacities of the bowling and fielding team by sharing current evidence-based inputs gathered through search engines. This considerably helped the offline treating team (the bowlers and fielders) to intensify their strategy to finally get all the batsmen out. This patient’s course was such that one batsman after another tried to score as much as possible, as once she appeared to have recovered from the sepsis, she developed a myocardial infarction. Once that was out, she developed recurrent seizures, which were possibly a result of her uremic encephalopathy. She finally recovered, with all the wickets taken. A graphical representation of the entire hospital course of her illness is shared (Figure 5), here.

### 3.2. Precision Medicine through a Neurologist’s Narrative Lens (in Relatively High-Resource Settings)

KS has been a member of our CBBLE since 2006, when RB, his offline teacher in his medical college in Bangalore migrated on a teaching assignment to Malaysia and he chose to keep in touch online. Since then, he has completed his postgraduate residency in medicine and fellowship in neurology. He currently practices interventional neurology in a high-resource setting in India, where he often works in the neuro cath lab performing angiography and stenting of cerebral blood vessels. What follows is a case-based narrative in his voice.

A 29-year-old male, presented to our Neurology Clinic with his first known episode of generalized tonic-clonic seizures. He denies drug or alcohol usage and his growth and developmental history was normal. Prior to this hospital visit, he had not attended any medical consultation. He was not on any medications for any other ailment. His neurological examination was normal. He was then subjected to an electroencephalography (EEG) and magnetic resonance (MR)imaging of the brain. EEG was normal. However, MR imaging (with contrast) revealed a single ring-enhancing lesion in the left temporal lobe with a visible scolex. A diagnosis of Neurocysticercosis was made. The patient was prescribed Albendazole and steroids for the acute problem and then prescribed T. Levetiracetam 500 mg twice a day for the seizures.

Two weeks into treatment he was brought to the emergency room (ER) in an unresponsive state. His parents found him unresponsive and noticed that he had inadvertently passed urine in his clothes. On examination, his vitals were stable. He had a gaze preference to the left, with paucity of movements on the right side. The possibility of an unwitnessed seizure, Todd’s palsy, and postictal confusion were suspected. A repeat MR imaging revealed acute infarct in the left middle cerebral artery (MCA) and left anterior cerebral artery (ACA) territories. MR angiogram revealed a left carotid occlusion. His older MR images were reviewed and showed no evidence of carotid or intracranial vascular disease. He was transferred to the angiography suite and a diagnostic cerebral angiogram was performed. Digital subtraction angiography (DSA) revealed a left carotid T occlusion. A mechanical thrombectomy was successfully performed. However, there was also evidence of left MCA occlusion. Mechanical thrombectomy was repeated, with partial recanalization. Repeat brain imaging revealed significant. Left hemispheric infarct with evidence of evolving cerebral edema. A decompressive craniectomy was done 3 days after the surgery, repeat imaging revealed persistent cerebral edema and hence he was referred for re-exploration. He underwent left frontal and temporal lobectomy. The patient is slowly recovering with occupational and rehabilitative therapies.

A young stroke workup was considered. Routine blood investigations were normal. Autoimmune markers including antinuclear antibody (ANA) and antineutrophil cytoplasmic antibodies (ANCA) were normal. He had normal homocysteine levels and a normal echocardiogram. Holter monitoring was normal. We screened him for genetic thrombophilic conditions. He was found to have a homozygous MTHFR C677T gene mutation. MTHFR mutation has been previously linked to ischemic strokes.

Two hit hypothesis is well discussed in the field of oncology where a reactive genetic mutation can be triggered by an epigenetic event such as an infection. However, if we are to apply the same principle in this case scenario, our patient had a ‘first hit’ with a homozygous MTHFR C677T mutation. The ‘second hit’ may have been the neurocysticercosis infection, followed by the interventions or the resultant systemic distress and functional recovery.

#### Analogy-Driven Analysis: Retrospective Notes from the Dugout

In the above game, the bowling and fielding team of KS played with the best possible strategy (precision), but was unable to get the disease out (accuracy), and the game appeared to be drawn, if not lost.

### 3.3. Precision Medicine (in Relatively Low-Resource Settings) through the Lens of a Medical Student’s Online Interaction with Our CBBLE

VP is a medical student from Tairunnessa Memorial Medical College, Gazipur, Bangladesh and an active member of our CBBLE who has attended the offline component of our CBBLE, which is also organized in collaboration with BMJ case reports [22]. After finishing his offline elective stint a year back in our host Institute, he has subsequently remained active online and regularly collates patient data shared by different members of our CBBLE into deidentified online patient records and ensures that all the signed informed consents from these patients are maintained and preserved in secure locations. The physicians in our CBBLE share their patient data in the hope of obtaining online feedback through conversational engagement with peers along with current best evidence support which VP provides through extensive searches of online and offline electronic resources. The patient narrative below was posted on our WhatsApp discussion forum by a CBBLE member and collated by VP.

A 52 year old woman, who was visiting us from North Bengal said she was watching TV in her apartment one day in the summer of 2015 and suddenly noticed that the TV was swinging followed by swinging walls and even the stairs of her apartment when she quickly started running down to safety and amidst all this commotion she noticed a buzz of people shouting earthquake! Earthquake!! She remembers that is the first time she had experienced severe palpitations since then which she complains of repeatedly experiencing over the years. However, she didn’t notice the swelling in front of her neck that we noticed. We didn’t notice any protruding eyes or tremors etc. USG neck showed nodules in the thyroid that was sent for fine-needle aspiration cytology (FNAC) and her T4 was double the normal value and TSH was very low.

Toxic adenoma is a toxic thyroid nodule which produces excessive thyroid hormones. They are managed either by prolonged thionamide therapy, surgery or radioiodine ablation. In this non-diabetic patient, a non-velvety, hyperpigmented area involving skin folds was noticed at the back of the neck that was very atypical of acanthosis nigricans. In view of thyroid nodule and hyperpigmentation in the neck, paraneoplastic acanthosis nigricans was brought into consideration. Later, FNAC report was available which showed features of benign thyroid lesion with foci of mild atypia.

**Figure 6:**
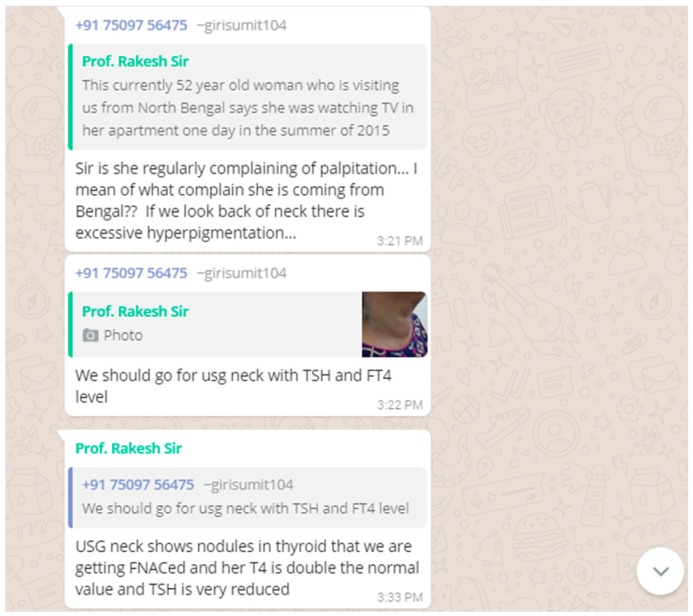
Illustrates the feedback and the queries posted from the CBBLE online network.

Once the above narrative was posted by one of our CBBLE users feedback started pouring in, in the form of queries and clarifications sought (Figure 6).

#### 3.3.1. After the Discussion, the Following Questions about the Patient Were Raised

If FNAC is positive, we would know what to do next, but what are the chances of false negativity after FNAC? What is the frequency of having to obtain excision/incision biopsies in such situations? What if we miss an underlying malignancy and go for radioiodine? Based on FNAC findings, how would you decide between surgery and medical management in this patient?

These were answered by VP after searching online and offline resources and illustrated by him below along with interactions with the initial CBBLE user who had posted the patient data.

#### 3.3.2. If FNAC Is Positive, We Would Know What to Do Next, But What Are the Chances of False Negativity after FNAC?

This made us look up specificity and sensitivity of FNAC in detecting thyroid malignancy. A study showed FNAC is more specific (98%) than sensitive (80%) in detecting malignancy [23]. Here if FNAC is more specific than sensitive so what should we do if the FNAC is negative? There is still 20% chance of its being malignant.

#### 3.3.3. What Is the Frequency of Having to Obtain Excision/Incision Biopsies in Such Situations?

*The decision to go for excision biopsy primarily for histopathological examination (HPE) to rule out malignancy is often a judgement call. In this context from a precision medicine perspective would a liquid biopsy be feasible or help? A feasibility study was conducted by detecting BRAF(V600E) circulating tumour DNA (ctDNA) in the plasma of patients with thyroid nodules to distinguish between benign and malignant nodules. Results showed that BRAF(V600E) ctDNA could distinguish between the two* [24].

#### 3.3.4. Based on FNAC Findings, How Would You Decide between Surgery and Medical Management in This Patient?

The problem with both radioiodine and surgery is that the patient would become hypothyroid eventually and end up in a lifelong dose of maintenance thyroxine. The problem with carbimazole is that we may not be sure of the response.

Eventually, the patient was started on carbimazole after shared decision making with her and her relatives.

#### 3.3.5. Analogy-Driven Analysis: Retrospective Notes from the Dugout

This game involved a fair amount of precision even in a relatively low-resource setting. It appears to have achieved a fair amount of accuracy, although the game is going to be long, as thyroid-suppressive treatment takes a long time to maintain remission in toxic adenomas. There is always a chance of a malignancy showing up sometime later. This patient was actually referred to our CBBLE from a very distant community, nearly 2000 km away from our tertiary care teaching hospital. VP and RB were already evaluating the patient’s inputs posted by one our CBBLE community health workers, who also resides in the same community 2000 km away, in the patient’s village/town. A tentative plan for evaluation was made online, following which the patient was further evaluated in the hospital after she made the long journey. The revised inputs on the patient after evaluation by the hospital residents and faculty were again posted to the online network on WhatsApp, and a final plan of available intervention was drawn. There was a question raised by the team evaluating the patient in the hospital about the uncertainty of future malignancy prediction in this patient when an online CBBLE member suggested that this could become feasible in the near future using BRAF (V600E) ctDNA. At the current point in time, this is out of reach from a rural Indian tertiary teaching hospital perspective, but it is possible that resources and research could make this available even in less than a decade, in the coming years. Until then, we may have to live with the uncertainty posed.

### 3.4. Precision Medicine through a Hematoncologists Narrative Lens (in Relatively High-Resource Settings)

Author BD is one of the earliest students of our CBBLE from the purely email era, when he published some of his cases even as a graduate student in Manipal College of Medical Sciences, Pokhara, Nepal, way back in 2002. He is currently working at the cutting edge of precision medicine in a high-resource setting at the Medical College of Wisconsin, as a hemat-oncologist focused on multiple myeloma and other plasma cell disorders.

In a frigid Wisconsin morning last December, I received a phone call from my friend, whose brother was in the battle for his life against Multiple Myeloma. This patient’s disease had progressed after multiple lines of chemotherapy including two stem cell transplants in the span of 4 years. My friend was desperate for a ray of hope to help his brother. He had exhausted all available options, and enrollment in a clinical trial was the only potential solution for his disease. As a myeloma focussed physician, I was aware of a clinical trial that was exploring an experimental agent for the subtype of myeloma he had; myeloma with chromosomal translocation 11 and 14; t(11:14). With ample caution, I routed them towards relevant myeloma clinical trials in their area. Fast forward a few months, my friend called me again, but this time excitement was palpable through the phone. His brother was enrolled in a clinical trial using bcl 2-inhibitor (venetoclax)—a drug active against a particular subtype of multiple myeloma with t(11:14) [25]. This result, a remission, was shared by others and this would open a new era of precision medicine in the therapeutic armamentarium of multiple myeloma.

#### Analogy-Driven Analysis: Retrospective Notes from the Dugout

The patient’s disease was difficult to drive out in spite of the best possible efforts by the fielding team over 4 years, and a chemical that targeted a precise molecular pathway in the pathogenesis of the disease offered promise of finally making a dent in the disease.

## 4. Discussion

### 4.1. Current Narratives in Precision Oncogenomics

In 1927, a brilliant physicist from Germany, Werner Heisenberg, introduced a principle, well known as “The Heisenberg Uncertainty Principle”, which would become pivotal for the development of quantum mechanics. The principle asserts the fundamental limit to the precision with which certain pairs of physical properties of a particle can be known. Modern medicine, particularly within the field of oncology, is rapidly moving towards a precision approach, as the molecular underpinnings of disease evolution are made known. As one of the major goals of cancer research is the gaining of understanding of the genetic changes responsible for the establishment of the “cancer clone” and the “key pathways”, they could be targeted therapeutically. New insights into human cancers are emerging from basic research, and this has the potential to augment disease diagnostics, therapeutics, and clinical decision-making.

“Precision Medicine”—an abundant term in medical literature—refers to the tailoring of medical treatment guided by genomic or molecular features of the disease and not by the clinicopathological features [26]. Since cancer is a disease of the genome, the field has been the perfect choice to enhance the impact of precision medicine [27]. Because every single cancer patient exhibits a different genetic profile, and that profile can change over time, “tailored” treatment, rather than a “one-size-fits-all” approach, is likely to benefit patients, and hence is an attractive concept. Whether it is a mere concept or realistically assures a better future in oncology continues to remain a debate. The precision medicine approach has transformed the outlook for some deadly cancers. One of the most notable examples is the discovery of bcr-abl gene fusion and the development of imatinib for Chronic Myelogenous Leukemia (CML). CML treated by imatinib resulted in unprecedented results, with a 5-year survival of 90% and some patients even inching towards cure [28]. Other examples include the human epidermal growth factor receptor-2 (HER-2) and development of agents like trastuzumab. Compared to conventional chemotherapy, the addition of this agent has resulted in significant improvement in progression-free survival and reduction of death by 20% [29]. These examples illustrate how the identification of key molecular pathways targeted therapeutically could alter the disease course and result in the desired outcomes. What about other cancers? Has precision medicine delivered its promise in other cancers, as well, and is it a time to celebrate? Or has our approach for more “precision” resulted in more “uncertainty”, as described by Heisenberg?

In this context, the design and the results of the SHIVA trial are worth a discussion. It is a phase 2, randomized multicenter trial, which assessed the efficacy of several molecularly targeted therapies based on molecular profiling compared to conventional therapies in patients with advanced cancers [30]. The results showed no improvement in progression-free survival (the primary end-point) with the use of molecularly targeted agents compared to physician’s choice of chemotherapy. However, it is important to realize that the majority of the patients in the trial received a hormone modulator or mTOR inhibitor, and thus the justification of the failure of the precision approach based on this limited data is not reasonable. The other was that the study was powered to determine whether the use of an algorithm-based approach to treatment allocation can improve patient outcomes—regardless of the nature of such allocated treatments [30]. In the trial, each patient served as his or her own control in terms of primary end-point assessment. This calls for novel methods of designing the trials, with clinically meaningful endpoints using precision medicine. One of the other concerns has been the possible lack of valid biomarkers. We are experiencing another revolution in personalized treatment, with the introduction of various immune-based approaches. Cancer immunotherapy used to have limited applications, mainly for selected cancers like melanoma and renal cancers and involved the use of interleukin 2 (IL-2). With the dramatic progress in the last few years, this approach has moved into the mainstream in several cancers. Currently, immune-based approaches represent the most exciting area for diseases like melanoma, non-small lung cell cancer, and hematological cancers including multiple myeloma. The immune approach includes both active and passive immunotherapies: monoclonal antibodies, checkpoint inhibitors, and cellular immunotherapy. Recently, Rosenberg et al. demonstrated a new approach to immunotherapy, in which “adoptive transfer of mutant-protein-specific tumour-infiltrating lymphocytes (TILs) in conjunction with interleukin (IL)-2 and checkpoint blockade mediated the complete durable regression of metastatic breast cancer, which is now ongoing for >22 months” [31]. However, not all patients respond to immunotherapy, and there is a variability in response. One of the reasons for the variability in patient response with immunotherapy is the lack of predictive biomarkers. Identifying predictive biomarkers is a challenge in immunotherapy, and the other challenges include cost, toxicity and tumor heterogeneity, which impede the efficacy of immune-based therapy. For checkpoint inhibition, PDL1 has been proposed as a putative biomarker, as pembrolizumab (anti-PD 1) is approved in non-small-cell lung carcinoma (NSCLC) only in patients whose tumor PD-L1 levels are ≥50% [32]. Unfortunately, with the different temporal, spatial and methodological heterogeneity, it remains an unreliable biomarker [27]. The other unreliable biomarker includes the ERCC1 for NSCLC to platinum therapy [33].

Despite the challenges, precision medicine holds a lot of potential in cancer therapy. While we need to conduct well-designed randomized trials to assess broader efficacy of personalized medicine and validate the bio-markers, we can also enjoy the tremendous success of KIT mutations in gastrointestinal stromal tumor (GIST), BRAF (V600E) in melanoma, EGFR, ALK, and ROS1 alterations in NSCLC [34,35,36]. In a study in which my friend’s brother participated, venetoclax monotherapy resulted in unprecedented response rates of 40% in heavily pre-treated patients with multiple myeloma [25]. Biomarker analysis confirmed that response to venetoclax correlated with higher BCL2:BCL2L1 and BCL2:MCL1 mRNA expression ratios, which are predominantly seen in patients with t (11:14). These outcomes show potential in results, for the first time, that pave the way for precision medicine in multiple myeloma with a significant potential to change practice in specific subgroups of patients and the hope is that knowledge in the field will increase to include other subgroups.

Clinical trials are evolving to investigate tumor heterogeneity from patient to patient in their design. The Molecular Analysis for Therapy Choice (NCI-MATCH) is a clinical trial selecting treatments based on genetic features of patients, not traditional tumor histology [32]. Thusfar, 2500 patients in the USA have been enrolled in one of the 24 arms of this trial, representing one half of the recruitment goals [37]. The Molecular Profiling-based Assignment of Cancer Therapy (NCI-MPACT) is another innovative clinical trial to test the hypothesis that targeting an oncogenic driver mutation is more efficacious than not targeting it. NCI-MPACT will recruit advanced cancer patients who have been unresponsive to standard therapeutic options and possess mutations in one of three genetic pathways that include DNA repair, PI3K/mTOR (phosphoinositide-3 kinase/mammalian target of rapamycin), and Ras/Raf/MEK (mitogen-activated protein kinase). The efficacy of diagnosis and therapies using precision medicine could be significantly enhanced, should results deliver the outcomes investigated in these trials.

With the development of novel technologies, it is hoped that understanding of tumor complexity and the immune system will be increased. These will be critical in designing future tailored combination therapies. The recent advances in the development of sequencing technologies have enhanced the ability to sequence cancers at both population and single-cell levels. Diverse mechanisms that lead to disease evolution, disease response, and refractoriness are slowly being understood. These advancements, we hope, will translate to more targeted therapies with better outcomes in patients with cancers in future.

The above discussion by author BD perhaps reflects and echoes the thoughts of many of his peers, who are working to make the dream of precision medicine a reality; while at the same time, this is an evolving area, where there are unseen threats (for patients) and opportunities (for pharma) in terms of aggravating the pandemic of overdiagnosis and overtreatment [38,39,40].

### 4.2. Age-Old Precision Medicine

The patient of KS presented to a relatively high-resource set-up in India, where the practitioner worked in a highly specialized and narrow domain of precision medicine (interventional neurology) and made the best precise efforts to address the problem in this critically ill patient, but the results were far from accurate. We are not sure of the outcomes that may have been achieved if the same patient had visited a low-resource setting; would the approach have been imprecise but the outcomes serendipitously better? Currently, what are the available strategies for predicting patient outcomes in response to highly specialized precision intervention? Randomized controlled trials have looked at catheter-based interventions in cerebral vessels, and available data supports the use of mechanical thrombectomy from 6 to 24 h for patients with occlusion of the intracranial carotid or proximal middle cerebral artery who present to a stroke center with expertise in both mechanical thrombectomy and automated infarct volume determination using MR imaging or perfusion CT [41]. While the data continues to evolve, there shall remain a grey zone where the push of newer innovations in terms of pharmacological and non-pharmacological devices will encourage overdiagnosis and overtreatment. This appears directly reactionary to combat pre-existing underdiagnosis and undertreatment, particularly in parts of the globe that are plagued by it.

The two patient narratives from relatively low-resource settings naturally reflect the danger of underdiagnosis and undertreatment. This is apparent beginning with the very first narrative, where the urinary tract infection was undertreated due to underdiagnosis. This was again a result of informational discontinuity. This is another malady that our CBBLE is striving to address by tracking each patient record online and encouraging physicians to communicate around their online patient records, which can be accessed through our CBBLE. The solution to this lies in concerted team communication. Ideally, if this patient had first approached our CBBLE, we could have utilized our online network, connected to microbiologists, to collect her results. While this is easier in patients admitted to our hospital, the same may not be true with outpatients. For such situations, we have in the past tried to train community health workers to follow up with our outpatients even after they return to their homes. We have tried to integrate this with rudimentary home health care programs, but we must admit that we have not been able to scale this approach beyond just one or two towns in rural India [42].

Identification, elucidation and correlation of external life event pathways through traditional history taking and internal cellular and molecular event pathways through current precision medicine approaches could serve as a superior predictor of outcomes than the binary preference-bound “patient-related outcome”. It can begin with recording and documentation of individual case-based experiences of the external and internal pathways that are otherwise routinely lost to science, and all this data can be harnessed using case-based reasoning (CBR) techniques. Many labels, such as case-based informatics and evidence farming, have been applied to these currently fledgling endeavors [43,44,45,46]. Our elective students have begun the process through their case records in their online learning portfolios [47], and in the next few months to years, once the number of cases in our database improves, we shall be able to utilize algorithms for “experiential evidence farming” to demonstrate improved patient outcomes with recording, sharing, reusing and recycling steps in the CBR evidence farming cycle.

## 5. Conclusions

The above paper was written to illustrate how CBBLE can strengthen the bridge between age-old precision approaches with modern omics-driven approaches to deliver precision healthcare and reduce overdiagnosis and overtreatment. The narratives from our CBBLE found that physicians naturally aim for precision and accuracy, although accuracy may remain elusive in spite of our best focused precise approaches. One can considerably increase one’s chances of obtaining accuracy if one is regularly supported by a CBBLE that not only offers evidence-based decision tools, but also a regular connection with peers that can go a long way toward improved documentation, transparency and newer learning insights.

We hope to scale the CBBLE approach in the near future to not only reduce overdiagnosis and overtreatment, but also promote transparency, accountability, and innovation toward optimizing solutions for individual patients, integrating age-old as well as technology-driven precision medicine approaches.
